# S100A4 Is Critical for a Mouse Model of Allergic Asthma by Impacting Mast Cell Activation

**DOI:** 10.3389/fimmu.2021.692733

**Published:** 2021-07-22

**Authors:** Tongqian Wu, Lan Ma, Xiaoqian Jin, Jingjing He, Ke Chen, Dingshan Zhang, Rui Yuan, Jun Yang, Qin Zhong, Haiyan Zhou, Zou Xiang, Yu Fang

**Affiliations:** ^1^ Center for Clinical Laboratory, Affiliated Hospital of Guizhou Medical University, Guiyang, China; ^2^ School for Clinical Laboratory, Guizhou Medical University, Guiyang, China; ^3^ Center for Pediatric Medicine, Affiliated Hospital of Guizhou Medical University, Guiyang, China; ^4^ Department of Health Technology and Informatics, Faculty of Health and Social Sciences, The Hong Kong Polytechnic University, Hong Kong, China

**Keywords:** S100A4, mast cell, allergic asthma, airway inflammation, allergy

## Abstract

**Background:**

The calcium-binding protein S100A4 demonstrates important regulatory roles in many biological processes including tumorigenesis and inflammatory disorders such as allergy. However, the specific mechanism of the contribution of S100A4 to allergic diseases awaits further clarification.

**Objective:**

To address the effect of S100A4 on the regulation of mast cell activation and its impact on allergy.

**Methods:**

Bone marrow-derived cultured mast cells (BMMCs) were derived from wild-type (WT) or S100A4^-/-^ mice for *in vitro* investigation. WT and S100A4^-/-^ mice were induced to develop a passive cutaneous anaphylaxis (PCA) model, a passive systemic anaphylaxis (PSA) model, and an ovalbumin (OVA)-mediated mouse asthma model.

**Results:**

Following OVA/alum-based sensitization and provocation, S100A4^-/-^ mice demonstrated overall suppressed levels of serum anti-OVA IgE and IgG antibodies and proinflammatory cytokines in serum, bronchoalveolar lavage fluid (BALF), and lung exudates. S100A4^-/-^ mice exhibited less severe asthma signs which included inflammatory cell infiltration in the lung tissue and BALF, and suppressed mast cell recruitment in the lungs. Reduced levels of antigen reencounter-induced splenocyte proliferation *in vitro* were recorded in splenocytes from OVA-sensitized and challenged mice that lacked S100A4^-/-^. Furthermore, deficiency in the S100A4 gene could dampen mast cell activation both *in vitro* and *in vivo*, evidenced by reduced β-hexosaminidase release and compromised PCA and PSA reaction. We also provided evidence supporting the expression of S100A4 by mast cells.

**Conclusion:**

S100A4 is required for mast cell functional activation, and S100A4 may participate in the regulation of allergic responses at least partly through regulating the activation of mast cells.

## Introduction

Allergy is one of the most prevalent human diseases affecting the quality of life of about 20% of the global population, the incidence of which has been steadily increasing over the past decades. This increasing trend is particularly notable for allergic asthma and rhinitis, which has led to an enormous socio-economic burden worldwide (1). Currently more than 300 million people suffer from asthma globally, and this number may likely be increased by another one third by 2025 (2). Innovative asthma and allergy treatment approaches to tackle this chronic disease are needed (3). As a complex disease, the pathogenesis of asthma is associated with the interaction of more than 100 genetic sites and complex environmental factors (4).

Allergic asthma is a typical type-2 helper T cell (Th2)-mediated chronic inflammatory disease of the airways (5). The lung tissue and bronchi are the main effector sites of allergic asthma. Classically, the mast cell is defined as a critical regulator and effector in the development and exacerbation of allergic pathology because of their potential to secrete a variety of allergic mediators (6). Mast cells are derived from multipotent hematopoietic progenitors in the bone marrow. Subsequently, mast cell progenitors migrate to various vascularized tissues, where they mature and differentiate into different phenotypes in response to local microenvironmental stimuli (7). Increased numbers of mast cells are observed at sites of allergic inflammation, and the mast cell density in the tissue correlates with the severity of allergic symptoms (8). In allergy, plurivalent antigens bind and crosslink IgE molecules bound to the high-affinity IgE-receptor (FcεRI) expressed on mast cells, resulting in cell degranulation and the release of allergic mediators including a huge spectrum of proinflammatory cytokines and chemokines. IgE-mediated mast cell degranulation initiates the early phase of an allergic response, resulting in pathologies, including increased epithelial permeability, mucous production, smooth muscle contraction, vasodilatation and neurogenic inflammation. The immediate response is followed by further production and secretion of Th2 cytokines, including IL-4, IL-5, and IL-13, as well as chemokines, that are responsible for the recruitment of various other immune effector cells, which participate in the late phase of the reaction, resulting in further exacerbation of allergic pathology (8, 9).

S100A4 is a member of the S100 protein family composed of a group of about 20 structurally related calcium-binding proteins (10). One of the family members, S100A4, has been reported to regulate a diverse range of cellular processes that affect the growth, survival, differentiation and motility of many cell types (11). S100A4 is expressed broadly in various cells, including fibroblasts, endothelial cells, smooth muscle cells, lymphocytes, neutrophils and macrophages (12, 13). This protein has no identified enzymatic function and it is predicted to exert its biological effects through interactions with several target proteins (14). S100A4 interacts with cellular targets through at least two receptors: the receptor for advanced glycation end-products (RAGE) and toll-like receptor 4 (TLR4) (15). S100A4 is an evolutionarily conserved protein and its human and mouse forms share 93% sequence identity. Some monoclonal antibody clones raised against human S100A4 also recognize mouse S100A4 (16). Furthermore, we have previously demonstrated that human S100A4 can regulate mouse leukocytes including dendritic cells and T cells (17).

Clinically oriented research on S100A4 has largely focused on the cancer metastasis-promoting properties of this protein (10). However, studies of the roles of S100A4 in non−tumor pathophysiologies are emerging. S100A4 has been shown to promote pathological inflammatory conditions, including rheumatoid arthritis, cardiovascular disease, fibrotic disease, and experimental autoimmune encephalomyelitis (11, 18–20).

We have previously provided compelling evidence demonstrating a clear contribution of S100A4 to allergy (17) and mucosal immune responses (21). We confirmed the impact of S100A4 on allergy using an experimental dermatitis model and a contact hypersensitivity model (17). However, the precise impact of S100A4 on mast cell-mediated allergic responses has not been clarified. In the current study, we demonstrate that S100A4 regulated mast cell activation and played a critical role in experimental models of asthma and anaphylaxis.

## Materials And Methods

### Animals

S100A4^+/+.GFP^ and S100A4^-/-.GFP^ mice, hereafter referred to as wild-type (WT) and S100A4^-/-^ mice, respectively, on a C57BL/6 background were obtained from Dr Zhihai Qin (20), and were bred in-house at the experimental animal facility of the Affiliated Hospital of Guizhou Medical University. Male mice that were 8-10 weeks old were used at the start of all experiments. All the mice were housed together under specific pathogen-free conditions at the experimental animal facility for at least 2 weeks before and for the duration of the experiments. The animal protocols were approved by the Ethics Committee of the Guizhou Medical University.

### Cell Culture

Bone marrow-derived cultured mast cells (BMMCs) were obtained by culturing bone marrow cells in RPMI 1640 medium containing 4 mM L-glutamine supplemented with 10% fetal bovine serum (FBS), 50 µM 2-mercaptoethanol, 1 mM sodium pyruvate, 0.1 mM MEM nonessential amino acids, 100 units/mL penicillin, and 0.1 mg/mL streptomycin (all from Sigma-Aldrich). The medium was further supplemented with 10 ng/ml rIL-3 (PeproTech) as a growth factor for murine mast cells. The cells were maintained at 37°C in 5% CO_2_ for 3 weeks and the purity of BMMCs reached 90%.

### Degranulation Assay

WT and S100A4^-/-^ BMMCs were pre-treated with 1 µg/mL anti-dinitrophenyl (DNP) IgE (clone SPE-7, Sigma-Aldrich) for 16 hours and were subsequently washed and challenged with 10 µg/mL DNP coupled to human serum albumin (HSA) with a coupling ratio between 30 and 40 (DNP-HSA; Sigma-Aldrich) for 30 minutes or 3 hours at 37°C in serum-free medium. The incubated supernatant for 30 minutes was collected for β-hexosaminidase measurement as a readout for mast cell degranulation, and incubated supernatant for 3 hours for cytokine measurement. Briefly, the supernatant was mixed with an identical volume of 4-nitrophenyl N-acetyl-b-D-glucosaminide (Sigma Aldrich), the substrate of β-hexosaminidase, and incubated for 1 h at 37°C. The reaction was stopped by the addition of an equal volume of 0.2 M glycine (Sigma-Aldrich) (pH10). The absorbance at 405 nm was measured using a microplate reader (22).

### Passive Cutaneous Anaphylaxis (PCA) Assay

The WT and S100A4^-/-^ mice were injected i.d. with 0.4 µg anti-DNP IgE directed against DNP in 10 µL PBS in the right ear, and an identical volume of PBS in the left ear. The passive cutaneous anaphylaxis reaction was induced 24 hours later by an intravenous injection of 10 µg DNP-HSA and 2% Evans blue in 200 µL of PBS. Mouse ears were removed 30 minutes later and the dye extravasation was quantified as previously described (22) with slight modifications. In brief, ears were ground by a tissue homogenizer with 1 mL PBS. The exudate was collected and mixed with acetone (3:7; v/v), and incubated at room temperature overnight. After vigorous vortexing, the mixture was centrifuged at 3000 rpm for 15 minutes. The supernatant was collected for the measurement of extravasated Evans blue with a spectrophotometer at 620 nm. For some of the PCA assays, Evans blue was obviated and ear tissues were sectioned and analyzed with toluidine blue staining for revealing mast cell morphology.

### Passive Systemic Anaphylaxis (PSA) Assay

The WT and S100A4^-/-^ mice were sensitized intravenously with 10 µg anti-DNP IgE in 200 µL of PBS, and the control groups were injected with an equal volume of PBS. Twenty-four hours later, the mice were challenged intravenously with 100 µg DNP-HSA, and core body temperature was recorded up to one hour after the provocation.

### Mouse Model of Allergic Asthma

WT and S100A4^-/-^ mice were sensitized by 4 intraperitoneal injections each with 20 μg OVA (grade V; Sigma Aldrich) admixed to 1 mg aluminium hydroxide (alum; InvivoGen) on days 0, 7, 14, and 21. On day 28 the mice were challenged by OVA aerosol (2%) in a container which was connected to a 403C air-compressing nebulizer (Yuwell) for 30 minutes daily for 7 consecutive days. The control mice received an identical volume of PBS for mock sensitization and challenge. Mice were killed 24 hours after the final challenge. Bronchoalveolar lavage fluid (BALF) was collected by flushing the lungs with 500 μL cold PBS. Serum was prepared through centrifugation and stored at -20°C for further analysis. The left lung lobes were removed and fixed in 4% formaldehyde overnight, and lobes of the right lung were processed for single cell preparation and flow cytometry analysis or stored at -80°C until further processing.

### Serological Analyses

Levels of OVA-specific IgG, IgG1, IgG2b, and IgE in serum were determined with the enzyme-linked immunosorbent assay (ELISA). Briefly, 96-well plates were coated with 200 μg/mL OVA and blocked with bovine serum albumin (1%; Sigma Aldrich). Serum samples were added at serial dilution followed by an overnight incubation at 4°C. Alkaline phosphatase-conjugated goat anti-mouse IgG, IgE, IgG1, or IgG2b (Southern Biotechnology Associates, Birmingham, AL, USA) was added (1:2000 dilution) and incubated for 3 hours. *p*-Nitrophenyl phosphate solution (Southern Biotechnology Associates) was used for color development, and absorbance at 405 nm was measured by a spectrophotometer. OVA-specific IgG, IgG1, IgG2b, and IgE titers were measured as previously reported (23).

### BALF and Lung Tissue Analyses

The total number of inflammatory cells present in BALF was determined using a haemocytometer. Cytospin centrifugation was performed followed by Wright’s staining for differential cell counting. At least 200 cells per sample were examined.

Lung tissue blocks were sectioned and stained with PE conjugated-cKit (Clone 2B8, Biolegend) and DAPI followed by examination using fluorescent microscopy for the expression of the fluorescent dyes including the cellular expression of GFP.

### Flow Cytometric Analyses

After tissue digestion and red blood cell lysis, single-cell suspensions were prepared from the lung tissues as reported (24). Cells were stained with the fluorescent-dye conjugated antibodies which included anti-CD11c (clone N418) and anti-F4/80 (clone BM8) from eBioscience, anti-CD3 (clone 17A2) and Siglec-F (clone E50-2440) from BD Biosciences, and anti-Gr1 (clone RB6-8C5) from ImmunoTools. Cellular production of S100A4 could be revealed by the expression of GFP in cells derived from the S100A4^+/+.GFP^ transgenic mice. Stained cells, together with the expression of GFP where appropriate, were analyzed using a flow cytometer (Navios; Beckman Coulter). Data were analyzed using FlowJo software (Tree Star Inc.).

### Antigen Recall Assay

Splenocytes (2.5 × 10^5^ per well) were plated into a 96-well round-bottom plate in the presence or absence of OVA (1 mg/mL) followed by culture at 37°C in 5% CO_2_ for 72 hours. The Cell Counting Kit-8 (CCK-8, Dojindo Molecular Technologies) was added to each well and the cells were incubated at 37°C in 5% CO_2_ for another 4 hours before being harvested for assessing the proliferative capacity of splenocytes (23). The culture supernatants were harvested for cytokine measurement.

### Pathological Analysis

The left lung lobes of each group were fixed by formaldehyde as described above. Next, the lobes were dehydrated, embedded in paraffin, and cut into 4-μm-thick paraffin sections. Hematoxylin-eosin staining was used to detect inflammatory cell infiltration, and pulmonary inflammation was evaluated by semi-quantitative evaluation of the cellular infiltrate defined as: 0 = no; 1 = slight; 2 = moderate; and 3 = abundant cellular infiltration as reported (23). The periodic acid-Schiff (PAS; Solarbio) staining was employed for detecting mucus secretion and goblet cells infiltration, and the scores were evaluated as follows: 0, <5%; 1, 5–25%; 2, 25–50%; 3, 50–75%; and 4, >75% (23). Meanwhile, toluidine blue was used to stain mast cells in the lung section, and mast cells were counted at least in three fields per section.

### Cytokine Measurement

Concentrations of murine IL-4, IL-5, IL-6, IL-10, IL-13, TNF-α, MCP-1 and IFN-γ in the BALF, serum, lung exudates, and splenocyte cultures were determined using a cytometric bead array assay (LEGENDplex™ Mouse Inflammation Panel, Biolegend) according to the manufacturer’s instructions. The fluorescence intensity was assessed on a Navios flow cytometer (Beckman, USA), followed by data analysis using LEGENDplex v8.0 software (Biolegend).

### Statistical Analysis

Data were analyzed using GraphPad Prism version 6.04 (GraphPad Software) and were presented as mean ± standard error of the mean. Statistical analysis was performed using a two-way ANOVA with Tukey multiple comparisons test. A *P* value < 0.05 is considered statistically significant.

## Results

### S100A4^-/-^ Mice Demonstrate Lower Levels of Humoral Immune Responses Following Allergic Sensitization and Asthmatic Challenge

**Figure 1:**
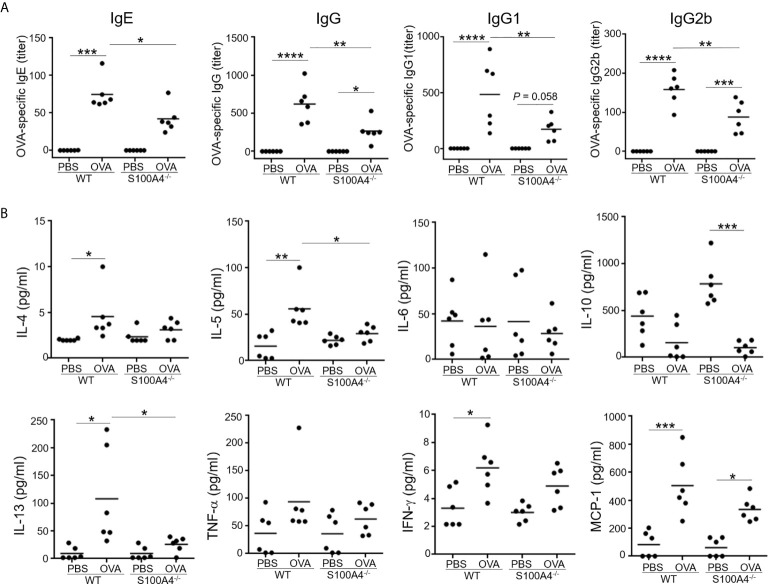
S100A4^-/-^ mice exhibit suppressed antigen-specific antibody and proinflammatory cytokine responses following asthmatic sensitization and provocation. Mice of wild-type (WT) and S100A4–/– strains were sensitized with 20 μg OVA adsorbed to 1 mg alum 4 times i.p. with a 1-week interval. Starting from day 28, mice were challenged with a daily exposure to aerosol of 2% OVA (w/v) for 30 minutes for 7 consecutive days. Control mice were administered with PBS on both occasions as mock immunization and provocation. Mice were killed one day after the last aerosol challenge, and serum was collected for analysis. **(A)** OVA-specific IgE, IgG, and IgG subclasses were measured using ELISA. **(B)** Relevant cytokines were analyzed using cytometric bead array analysis. Data are plotted where each dot represents the value of an individual mouse and the horizontal bars represent the mean. *P ≤ 0.05, **P ≤ 0.01, ***P ≤ 0.001, ****P ≤ 0.0001, using the two-way ANOVY with Tukey multiple comparisons test for statistical significance.

We have previously demonstrated the critical role of S100A4 in a skin dermatitis model and a contact hypersensitivity model (17). To further investigate the potential contribution of S100A4 to allergic asthma, we sensitized WT and S100A4^-/-^ mice, a different strain of the S100A4 knockout mice in contrast to the strain we used before (17), with OVA/alum followed by OVA aerosol challenge. Generation of allergen-specific antibodies including IgE is essential to the successful induction of the model. Therefore, we first analyzed mouse serum collected one day after OVA aerosol challenge for various types of OVA-specific antibodies. All WT asthmatic mice displayed increased levels of OVA-specific IgE and IgG as well as IgG subtypes compared with mice that only received PBS for mock sensitization and challenge. In contrast, despite the fact that S100A4-deficient mice also showed increases or a trend of increases in OVA-specific antibodies after provocation, their sensitization- and provocation-induced antibody enhancement was substantially lower than that observed in WT mice (**Figure 1A**).

We next analyzed a panel of molecules, including the Th2 cytokines IL-4, IL-5 and IL-13, the Th1 cytokine IFN-γ, the immune suppressive cytokine IL-10, the proinflammatory cytokine TNF-α, and the chemokine MCP-1, in the mouse serum. Sensitization and challenge augmented serum levels of IL-4, IL-5, IL-13, IFN-γ and MCP-1 in the WT mice, whereas these cytokines were either not upregulated or not to as high levels in S100A4^-/-^ mice (**Figure 1B**). IL-6 and TNF-α failed to show substantial regulation in this model in either the WT or S100A4^-/-^ mice (**Figure 1B**). There was a trend of increased levels of IL-10 expression in the control S100A4^-/-^ mice compared with the WT mice, suggesting that S100A4 might be able to suppress the constitutive expression levels of IL-10 which exerts immune suppressive function, thus favoring productive immune responses. Furthermore, IL-10 was found to be downregulated in S100A4^-/-^ mice after allergic sensitization and asthmatic challenge (**Figure 1B**), which may suggest a homeostatic role of S100A4 in maintaining immune vigilance.

### S100A4^-/-^ Mice Demonstrate Compromised BALF Responses After Allergic Sensitization and Asthmatic Challenge

**Figure 2:**
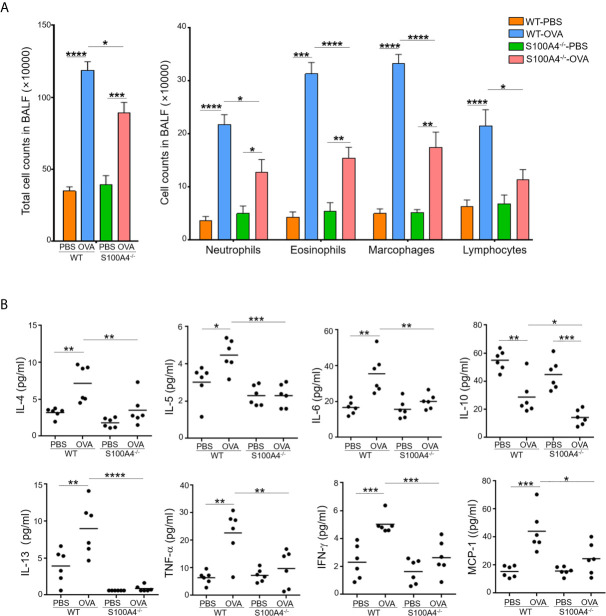
S100A4^-/-^ mice demonstrate reduced cellular infiltration and cytokine secretion in bronchoalveolar lavage fluid (BALF) following asthmatic sensitization and provocation. Mice of wild-type (WT) and S100A4^–/–^ strains were induced to develop experimental asthma as described in **Figure 1** and BALF was collected. **(A)** Total BALF cell number enumeration as well as cell type discrimination was carried out by Wright’s staining and microscopy. Data are presented as the mean ± SEM (n = 6). **(B)** Selected cytokines in BALF were analyzed by cytometric bead array analysis. Data are plotted where each dot represents the value of an individual mouse and the horizontal bars represent the mean. *P ≤ 0.05, **P ≤ 0.01, ***P ≤ 0.001, ****P ≤ 0.0001, using the two-way ANOVY with Tukey multiple comparisons test for statistical significance.

Cellular infiltration and proinflammatory cytokine production in the airway tissues is one of the hallmarks in the development of allergic asthma. We next analyzed cell infiltration and cytokine levels in the BALF. Mock-treated mice of both genotypes, WT or S100A4^-/-^, exhibited equally low level homeostatic responses with respect to total BALF cell numbers and differential cell counts including neutrophils, eosinophils, macrophages and lymphocytes. Upon allergic stimulation, WT mice experienced substantially enhanced cell infiltration in the BALF in terms of the total cell number and various types of the inflammatory cells (**Figure 2A**). The enhancement was consistently suppressed in S100A4-deficient mice (**Figure 2A**). Furthermore, we also evaluated the cytokine and chemokine responses in the recovered BALF as in serum. Levels of all the cytokines analyzed, except IL-10 which was downregulated, were upregulated after allergic provocation in the WT mice. In remarkable contrast, S100A4-deficient mice exhibited almost completely suppressed cytokine production after sensitization and challenge (**Figure 2B**).

### S100A4^-/-^ Mice Have Compromised Asthmatic Lung Responses After Allergic Sensitization and Asthmatic Challenge

**Figure 3:**
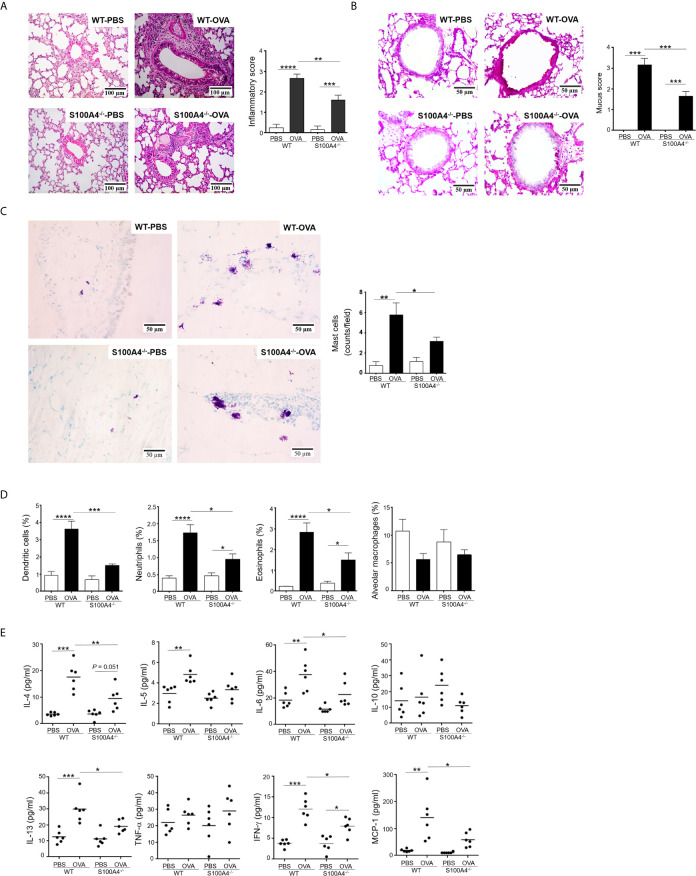
Cellular infiltration and cytokine secretion in lung tissues are suppressed in S100A4^-/-^ mice following asthmatic sensitization and provocation. Wild-type (WT) and S100A4^–/–^ strains of mice were induced to develop experimental asthma as described in **Figure 1** and lung tissue exudates were prepared. **(A)** Haematoxylin-eosin staining was performed and inflammation intensity was scored. **(B)** Periodic acid-Schiff (PAS) staining was used to reveal goblet cells and relative mucin production based on frequencies of goblet cells was estimated employing a semi-quantitative scoring system. **(C)** Mast cells were revealed by toluidine blue staining and counted (at least three random fields per tissue section). **(D)** Single cell suspensions from lung tissues were prepared for the assessment of infiltration of eosinophils (SiglecF^+^CD11c^–^), dendritic cells (SiglecF^–^CD11c^+^), neutrophils (Gr-1^+^CD11c^–^ SiglecF^–^), and alveolar macrophages (SiglecF^+^CD11c^+^F4/80^+^) by flow cytometric analysis. **(E)** Selected cytokines in lung tissue exudates were measured using cytometric bead array analysis. Data in **(A–D)** are presented as the mean ± SEM (n = 6). Data in **(E)** are plotted where each dot represents the value of an individual mouse and the horizontal bars represent the mean. *P ≤ 0.05, **P ≤ 0.01, ***P ≤ 0.001, ****P ≤ 0.0001, using the two-way ANOVY with Tukey multiple comparisons test for statistical significance.

The lung is the most important effector organ for asthma. Thus, we further examined the effect of S100A4 on lung inflammation. After asthmatic challenge, the lung tissues in the WT mice displayed pronounced infiltration of inflammatory cells, but the enhancement was compromised in the S100A4^-/-^ mice (**Figure 3A**). Challenge-induced mucin overproduction was also suppressed in the S100A4^-/-^ mice (**Figure 3B**).

### Lung Tissue Infiltration of Inflammatory Cells Including Mast Cells and Lung Cytokine Responses Are Suppressed in S100A4^-/-^ Mice After Allergic Sensitization and Asthmatic Challenge

Conventional laboratory mouse strains, such as the C57BL/6 used in this study, do not contain appreciable numbers of mast cells in lung parenchymal tissues (25). However, mast cell infiltration into the lung tissues can be induced after sensitization and provocation. We thus stained lung tissue sections with toluidine blue to reveal mast cell infiltration. Comparatively fewer mast cells were recruited in the asthmatic lung of S100A4-deficient mice compared with WT mice (**Figure 3C**). We next characterized infiltration of other inflammatory cells in the lung tissues *via* flow cytometry. Consistently, augmented infiltration of dendritic cells, neutrophils, and eosinophils was suppressed in S100A4-deficient mice compared with the WT mice following asthmatic challenge (**Figure 3D**). Asthmatic provocation failed to augment alveolar macrophage infiltration (**Figure 3D**).

To further address the inflammatory environment in the local lung tissues of the two mouse strains, we assessed the levels of inflammatory cytokines in the lung tissue homogenates. Of all the cytokines and chemokines analyzed, provocation-induced over production of IL-4, IL-6, IL-13, IFN-γ and MCP-1 was consistently suppressed in S100A4-deficient mice compared with the WT mice (**Figure 3E**).

### S100A4^-/-^ Mice Demonstrate Reduced T-Cell Memory Responses

**Figure 4:**
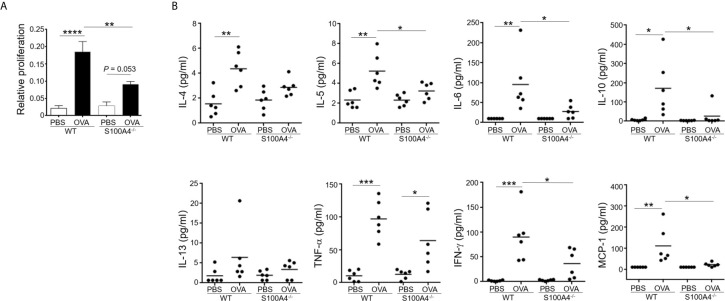
S100A4^-/-^ mice exhibit attenuated immune recall response following asthmatic sensitization and provocation. Wild-type (WT) and S100A4^–/–^ strains of mice were induced to develop experimental asthma as described in **Figure 1**. Splenocytes were harvested and incubated with 1mg/mL OVA for 3 days, and the CCK-8 reagent was added for the last 4 h. **(A)** Cell proliferation was measured using a spectrophotometry-based CCK-8 assay. Data are presented as the mean ± SEM (n = 6). **(B)** Supernatants were collected for the measurement of selected cytokines using cytometric bead array analysis. Data are plotted where each dot represents the value of an individual mouse and the horizontal bars represent the mean. *P ≤ 0.05, **P ≤ 0.01, ***P ≤ 0.001, ****P ≤ 0.0001, using the two-way ANOVY with Tukey multiple comparisons test for statistical significance.

Production of antigen-specific memory T-cells is crucial for the development of adaptive immune responses including allergy. We have previously shown that the T-cell memory response was suppressed using an allergic skin dermatitis model (17). We therefore would like to confirm the effect of S100A4 on this critical mechanism in the current mouse asthma model, which used an independent strain of the S100A4 knockout mice. To this end, we harvested mouse splenocytes, which contained abundant T cells and antigen presenting cells including dendritic cells, from mice that had sensitized and provocated with OVA. Antigen re-encounter-induced T-cell proliferation was assessed after the addition of OVA *in vitro*. Consistent with the previously described model (17), T cells from S100A4^-/-^ mice exhibited remarkably reduced antigen re-encounter-mediated proliferation compared with cells from the WT mice (**Figure 4A**). A consequence of the vigorous T cell memory response is the production of relevant cytokines. We next measured the cytokine levels in the supernatants of the splenocyte culture. Our data demonstrated compromised OVA-induced cytokine and chemokine production, including IL-5, IL-6, IL-10, IFN-γ and MCP-1, in the cultures of splenocytes from S100A4^-/-^ mice (**Figure 4B**). There was a trend of suppressed IL-4 and TNF-α increase in the S100A4^-/-^ cultures, albeit not reaching statistical significance (**Figure 4B**).

### Mast Cells Deficient in S100A4 Demonstrate Compromised IgE-Mediated Activation

**Figure 5:**
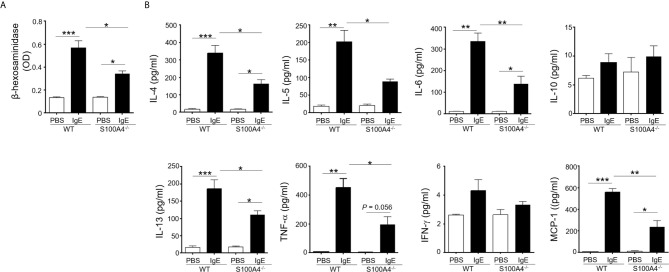
S100A4^-/-^ mast cells demonstrate compromised degranulation and cytokine production after IgE-mediated activation. BMMCs were cultured from wild-type (WT) or S100A4^-/-^ mice. Cells were pre-treated with 1 µg/ml anti-DNP IgE or vehicle control (PBS) overnight, followed by treatment with 100 µg/ml DNP-HSA. **(A)** Cell culture supernatant (serum-free) was collected 30 minutes after the addition of DNP-HSA. β-hexosaminidase release was measured using a colorimetric method and the results were shown as OD values. **(B)** Cell culture supernatant was collected 3 hours after the addition of DNP-HSA. Selected cytokines were analyzed by cytometric bead array analysis. Shown is one representative set of data of 3 experiments. Data are presented as the mean ± SEM (n = 3). *P ≤ 0.05, **P ≤ 0.01, ***P ≤ 0.001 using the two-way ANOVY with Tukey multiple comparisons test for statistical significance.

Mast cell activation has been documented as a major mechanism underlying allergic responses. In order to investigate the effect of S100A4 on mast cell activation, BMMCs cultured from mice sufficient or deficient in S100A4 were primed with anti-DNP IgE followed by incubation with DNP. BMMCs from S100A4 deficient mice exhibited an overall compromised degranulation and cytokine-production, evidenced by decreased β-hexosaminidase release (**Figure 5A**) and reduced levels of IL-4, IL-5, IL-6, IL-13, TNF-α and MCP-1 in the culture supernatants compared with cells from WT mice (**Figure 5B**). No statistically significant increases of IL-10 and IFN-γ levels were observed after antigen-reencounter-mediated cell activation and there were no apparent differences between the WT and S100A4^-/-^ cells (**Figure 5B**).

### Both PCA and PSA Responses Are Suppressed in S100A4^-/-^ Mice

**Figure 6:**
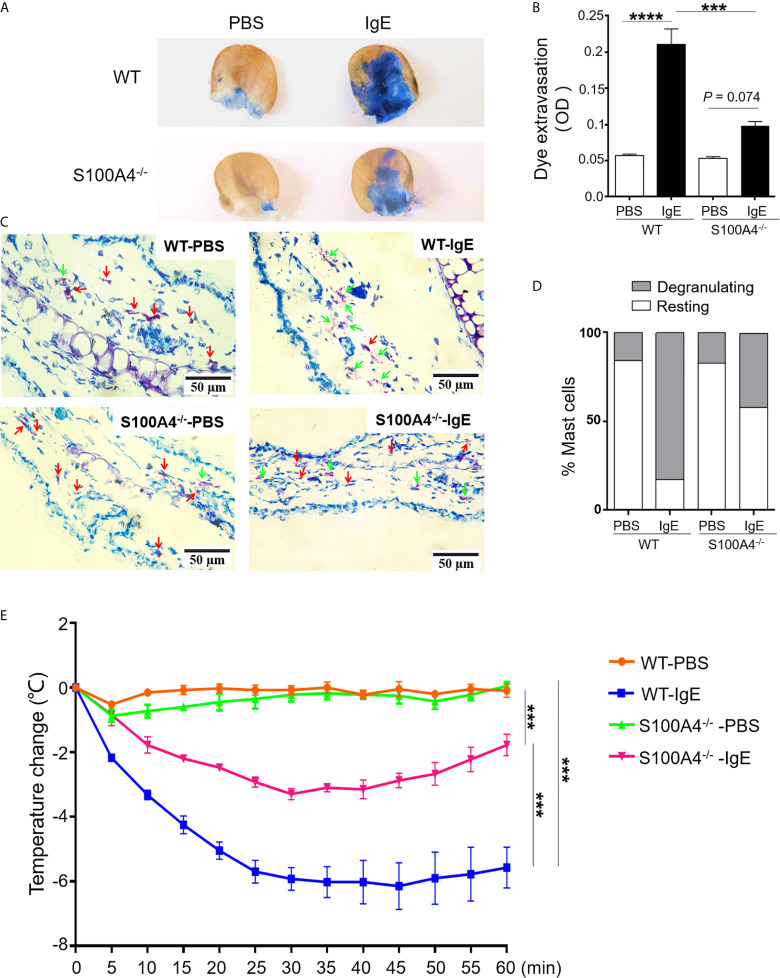
S100A4^-/-^ mice exhibit reduced passive cutaneous anaphylaxis (PCA) and passive systemic anaphylaxis (PSA). **(A–D)** Wild-type (WT) or S100A4^-/-^ mice were injected in the ear with anti-DNP IgE (right) or PBS (left) followed by a tail vein injection of DNP-HSA mixed with the Evans blue dye to induce the PCA reaction. The ears were collected 30 minutes later for the quantification of the tissue-extravasated dye, or sectioned to reveal mast cell morphology by toluidine blue staining. PCA reaction is demonstrated visually by dye accumulation in the ears **(A)** and quantification of the extravasated Evans blue dye in the ear by spectrophotometry **(B)**. Ear mast cell morphology was revealed by toluidine blue staining **(C)** and the numbers were quantified **(D)**. Red arrows, resting mast cells; green arrows, degranulating mast cells. **(E)** The WT and S100A4^-/-^ mice were sensitized intravenously with 200 µL of PBS alone or containing 10 µg anti-DNP IgE followed by intravenously administration with 100 µg DNP-HSA 24 h later, and core body temperature was recorded. Data in **(B, E)** are presented as the mean ± SEM (n = 6). ***P < 0.001, ****P ≤ 0.0001, respectively using the two-way ANOVY with Tukey multiple comparisons test for statistical significance.

In order to confirm the implication of S100A4 in mast cell activation *in vivo*, we assessed the intensities of PCA, a mast cell-dependent allergy model (26), in the WT and S100A4^-/-^ mice. Local IgE sensitization and systemic administration of DNP-HSA together with Evans blue triggered remarkable dye extravasation in the IgE-sensitized ear of the WT mice, and a substantially lowered level of dye extravasation was observed in the ear from the mice that lacked S100A4 (**Figures 6A, B**). Next, we tried to discriminate degranulating mast cells and resting mast cells based on the morphology of mast cell granules by toluidine blue staining as we previously reported (22). Consistent with the dye extravasation assay, we observed fewer degranulating mast cells in IgE-sensitized ears from the S100A4^-/-^ mice compared with WT controls (**Figures 6C, D**).

Although neutrophils have been described to be responsible for PSA involving allergen-reactive IgG (27), IgE-mediated PSA is mast cell-dependent (28). Systemic sensitization with anti-DNP IgE followed by intravenous challenge with DNP-HSA elicited a robust anaphylactic response in WT mice evidenced by a progressive temperature loss after allergen challenge (**Figure 6E**). In contrast, S100A4^-/-^ mice exhibited a less severe anaphylactic response after PSA provocation and started to recover from the temperature drop at about 30 minutes after the provocation, a time point when the low temperature of the WT mice was sustained (**Figure 6E**).

### Both *In Vitro* Cultured Mast Cells and Lung Tissue Mast Cells Express S100A4

**Figure 7:**
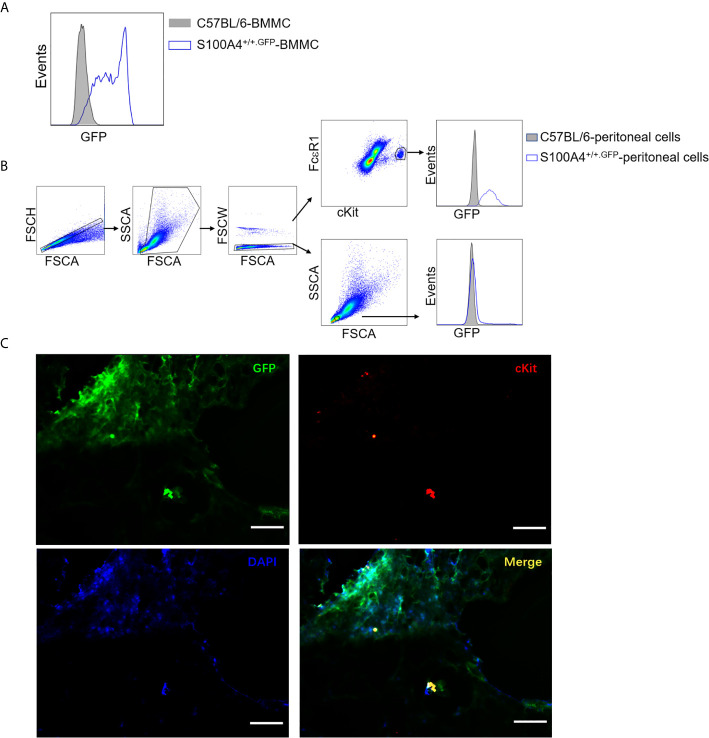
Mast cells express S100A4 and its deficiency does not affect mast cell differentiation. **(A)** BMMCs were cultured from non-GFP-containing normal C57BL/6 mice or S100A4^+/+.GFP^ mice. Expression of S100A4 by BMMCs was indicated by GFP expression after 3 weeks of culture. **(B)** Peritoneal cells obtained from normal C57BL/6 mice or S100A4^+/+.GFP^ transgenic mice were stained for FcεRI and cKit. FcεRI^+^cKit^+^ mast cells were gated for the analysis of GFP expression which indicates S100A4 expression using histograms. A population of FSC^low^SSC^low^ cells, which were negative for S100A4, were also gated for analysis as a negative control. **(C)** S100A4^+/+.GFP^ mice were sensitized with OVA/alum followed by challenge with OVA aerosol as explained in **Figure 1**. Lung tissue sections were prepared followed by staining with cKit and DAPI to show S100A4-expressing (GFP positive) mast cells (magnification, × 400; scale bar, 100 µm).

As the S100A4^+/+.GFP^ transgenic mice express green fluorescent protein (GFP) under control of the S100A4 promoter, we could determine whether mast cells expressed S100A4 by examining the GFP expression. GFP signal was clearly identified in cultured BMMCs (**Figure 7A**), mouse peritoneal mast cells (**Figure 7B**), and lung tissue mast cells in asthmatic mice (**Figure 7C**), thus confirming the expression of S100A4 by mouse mast cells. Prior to our study, Domenis et al. reported the expression of S100A4 by the human mast cell line HMC-1 and human tissue mast cells (29). The contribution of mast cell-associated S100A4 in allergic responses in humans await further investigation.

## Discussion

In addition to S100A4, the possible roles of other S100 family molecules in mast cell biology have been described. S100A6 suppress allergic inflammation by inhibiting the release of histamine from mast cells (30). At lower levels, S100A8 dampens mast cell activation and suppresses mast cell-mediated leukocyte adhesion and extravasation. However, at higher levels, S100A8 can directly activate mast cells and augment IgE-mediated mast cell activation (31). S100A12 is a chemotactic factor for mast cells (32) and promotes the release of pro-inflammatory cytokines from these cells (33). Despite the aforementioned regulation of mast cell responses by various S100 family members, the impact of mast cell-associated S100A4 on allergic pathology had not been addressed prior to our current work.

We previously revealed a critical role of S100A4 in allergy, based on using systems biology and both clinical and experimental validation approaches (17). We clearly demonstrated the important contribution to the development of allergy by S100A4 using mouse models of allergic dermatitis and contact hypersensitivity (17). In the present study, we extended this line of research by demonstrating the impact of S100A4 on allergy using a mouse asthma model, and revealing a possible role of S100A4 in mast cell functionality.

Our data demonstrated that airway inflammation, including inflammatory cell infiltration and cytokine production, typical features of allergic asthma, was suppressed in S100A4^-/-^ mice. As conventional laboratory mice, such as C56BL/6, do not have lung parenchymal mast cells, it is commonly believed that the acute mouse asthma model sensitized with OVA in the presence of alum as adjuvant followed by intranasal inoculation of OVA is not mast cell-dependent (25). Various approaches have been employed to induce the pulmonary recruitment of mast cells in mouse asthma models. An adjuvant-free sensitization protocol followed by chronic intranasal antigen challenge of mice results in the recruitment of mast cells in the lung tissue (34). In our asthma model, sensitization with OVA admixed to alum followed by exposure to consecutive challenges with aerosolized OVA spanning a week also induced lung recruitment of mast cells, which is consistent with a previous report (35). Interestingly, our data showed a marked suppression of mast cell recruitment into the lung tissues in the S100A4^-/-^ mice. The overall compromised asthmatic responses associated with the loss of S100A4 may therefore be accounted for, at least in part, by the fact that fewer mast cells were recruited into the lung tissues and a lowered level of mast cell activation as a result of S100A4 deficiency. Suppressed production of type-2 cytokines in the local lung tissues in the asthmatic mice deficient in S100A4 compared with the WT mice was consistent with reduced pulmonary recruitment of mast cells in the former. Furthermore, splenocytes from S100A4^-/-^ mice demonstrated reduced proliferation upon antigen re-encounter compared with those from WT mice, which suggests a contribution of S100A4 to the activation of T cell memory responses, which is consistent with our previous observations in an allergic dermatitis model (17) and a mucosal immunization model (21).

To demonstrate a clear involvement of S100A4 in mast cell biology, we cultured bone marrow-derived mast cells from mice bearing or lacking S100A4 and our data clearly demonstrated lowered responses of S100A4-deficient mast cells upon crosslinking of the IgE receptor by a model allergen, which is a classical mast cell activation pathway in allergic pathology. Mast cells lacking S100A4 responded with suppressed pre-formed granule-associated mediator release as well as *de novo* cytokine and chemokine production.

To further validate the *in vivo* relevance of mast cell-associated S100A4 in allergic pathology, we compared WT and S100A4^-/-^ mice in their responses to PCA and PSA reactions, models dependent on mast cell activation (36). Our data demonstrated drastically compromised anaphylactic responses induced in mice that lacked S100A4. This observation supports that, similarly as observed in BMMC, tissue mast cells deficient in S100A4 also have a defective capacity to launch the anaphylactic responses. Of course, we cannot rule out the possibility that the reduced intensities in the PCA and PSA models reflect the fact that the absence of S100A4 release from other cells and tissues in the S100A4^-/-^ mice may also contribute to reduced mast cell reactivity *in vivo*. Our present study demonstrates that this multi-functional molecule is likely to have an impact on allergy through the regulation of mast cell functionality. Other pathways relevant for allergic responses may also be regulated by S100A4. Of note, we have observed that antigen-reencounter-mediated production of IFN-γ by T cells was substantially suppressed in S100A4-defficient mice, which was consistent with a recent study which revealed an effect of S100A4 on regulating asthmatic responses through modulating T cell production of IFN-γ (Dr Zhihai Qin, personal communication).

## Data Availability Statement

The original contributions presented in the study are included in the article/supplementary material. Further inquiries can be directed to the corresponding authors.

## Ethics Statement

The animal study was reviewed and approved by Ethics Committee of the Guizhou Medical University.

## Author Contributions

TW performed the experiments and analyzed the data. LM and XJ were involved in the animal experiments. JH, KC, and DZ carried out the tissue analysis. HZ and QZ were involved in the *in vitro* cell analysis. RY and JY assisted with the data analysis and interpretation. TW, YF, and ZX wrote the manuscript. YF and ZX conceived the study. YF supervised the study. All authors contributed to the article and approved the submitted version.

## Funding

This work was supported by National Natural Science Foundation of China (Grant No. 81560266 and 81760294).

## Conflict of Interest

The authors declare that the research was conducted in the absence of any commercial or financial relationships that could be construed as a potential conflict of interest.
